# Cardiomyocyte tetrahydrobiopterin synthesis regulates fatty acid metabolism and susceptibility to ischaemia–reperfusion injury

**DOI:** 10.1113/EP090795

**Published:** 2023-05-15

**Authors:** Sandy M. Chu, Lisa C. Heather, Surawee Chuaiphichai, Thomas Nicol, Benjamin Wright, Matthieu Miossec, Jennifer K. Bendall, Gillian Douglas, Mark J. Crabtree, Keith M. Channon

**Affiliations:** ^1^ Division of Cardiovascular Medicine, British Heart Foundation Centre of Research Excellence, Radcliffe Department of Medicine University of Oxford Oxford UK; ^2^ Department of Physiology, Anatomy and Genetics University of Oxford Oxford UK; ^3^ Oxford Genomics Centre, Wellcome Centre for Human Genetics University of Oxford Oxford UK

**Keywords:** cardiac metabolism, ischaemia–reperfusion injury, tetrahydrobiopterin

## Abstract

Tetrahydrobiopterin (BH4) is an essential cofactor for nitric oxide (NO) synthases in which its production of NO is crucial for cardiac function. However, non‐canonical roles of BH4 have been discovered recently and the cell‐specific role of cardiomyocyte BH4 in cardiac function and metabolism remains to be elucidated. Therefore, we developed a novel mouse model of cardiomyocyte BH4 deficiency, by cardiomyocyte‐specific deletion of *Gch1*, which encodes guanosine triphosphate cyclohydrolase I, a required enzyme for *de novo* BH4 synthesis. Cardiomyocyte (cm)*Gch1* mRNA expression and BH4 levels from cm*Gch1* KO mice were significantly reduced compared to *Gch1^flox/flox^* (WT) littermates. Transcriptomic analyses and protein assays revealed downregulation of genes involved in fatty acid oxidation in cm*Gch1* KO hearts compared with WT, accompanied by increased triacylglycerol concentration within the myocardium. Deletion of cardiomyocyte BH4 did not alter basal cardiac function. However, the recovery of left ventricle function was improved in cm*Gch1* KO hearts when subjected to *ex vivo* ischaemia–reperfusion (IR) injury, with reduced infarct size compared to WT hearts. Metabolomic analyses of cardiac tissue after IR revealed that long‐chain fatty acids were increased in cm*Gch1* KO hearts compared to WT, whereas at 5 min reperfusion (post‐35 min ischaemia) fatty acid metabolite levels were higher in WT compared to cm*Gch1* KO hearts. These results indicate a new role for BH4 in cardiomyocyte fatty acid metabolism, such that reduction of cardiomyocyte BH4 confers a protective effect in response to cardiac IR injury. Manipulating cardiac metabolism via BH4 could play a therapeutic role in limiting IR injury.

## Abstract (highlights)

- **What is the central question of this study?**
  What are the physiological roles of cardiomyocyte‐derived tetrahydrobiopterin (BH4) in cardiac metabolism and stress response?
- **What is the main finding and its importance?**
  Cardiomyocyte BH4 has a physiological role in cardiac metabolism. There was a shift of substrate preference from fatty acid to glucose in hearts with targeted deletion of BH4 synthesis. The changes in fatty‐acid metabolic profile were associated with a protective effect in response to ischaemia–reperfusion (IR) injury, and reduced infarct size. Manipulating fatty acid metabolism via BH4 availability could play a therapeutic role in limiting IR injury.

## 1 INTRODUCTION

Generation of nitric oxide (NO) in the heart, by nitric oxide synthases (NOS), plays crucial roles in cardiac function and the response to cardiac injury (Schulz et al., 2004). The NO generation process requires the small molecule tetrahydrobiopterin (BH4). Loss of BH4 results in loss of NO generation and increased production of reactive oxygen species (ROS) by NOS, due to loss of enzymatic coupling between l‐arginine oxidation and reduction of molecular oxygen. We have previously shown, using cell specific knockouts of *Gch1*, encoding the required enzyme for BH4 synthesis, guanosine triphosphate cyclohydrolase I (GTPCH), that selective loss of endothelial cell BH4 in vivo induces endothelial NOS (eNOS) uncoupling with alterations in vascular NO and ROS signalling (Bendall et al., 2014; Douglas et al., 2018). Furthermore, we showed that augmentation of cardiomyocyte BH4, by targeted cardiomyocyte over‐expression of *Gch1*, selectively increases cardiomyocyte BH4 levels and augments myocardial NOS activity leading to alterations in myocardial relaxation, and protection against cardiomyopathy induced by diabetes (Carnicer Hijazo et al., 2021). However, the requirement for physiological *de novo* BH4 synthesis within cardiomyocytes remains unclear. Furthermore, BH4 may exert other redox effects on cellular metabolism via non‐canonical, NOS‐independent pathways that may be important in the response to myocardial ischaemia–reperfusion (IR) injury (Bailey et al., 2017).

High cardiac energy demand depends on ATP production with metabolic flexibility that can switch energy fuel source in response to different conditions. Under healthy conditions, fatty acids are the main source of myocardial energy supplying 60–70% of required ATP, as they are the most energy‐dense fuel (Kerr et al., 2017), but require greater oxygen consumption. Thus, under circumstances when oxygen supply is limited, such as in cardiac ischaemia, cardiomyocytes rapidly shift to anaerobic glycolysis in order to increase oxygen efficiency. The impact on metabolic reactions and changes in intracellular signalling pathways during cardiac ischaemia can lead to accumulation of long‐chain fatty acids and stimulation of ROS production (Liepinsh et al., 2013). Suppression of mitochondrial fatty acid oxidation can protect mitochondria from accumulating long‐chain fatty acid and preserve cardiac contractility and function post‐ischaemia (Johnston & Lewandowski, 1991; Liepinsh et al., 2013; Lopaschuk, 2000; Lopaschuk et al., 1990).

Previous studies have suggested that BH4 plays a role in substrate utilisation and cardiac metabolism. In diabetic hearts, increasing intracellular BH4 in cardiomyocytes elevates insulin‐independent glucose uptake and utilisation via neuronal NOS (nNOS)‐mediated pathways (Carnicer Hijazo et al., 2021). Therefore, BH4 could play a role in balancing and regulating cardiac metabolism in response to altered pathophysiological conditions. Furthermore, the availability of BH4 has been shown to modulate cardiovascular autonomic regulations in mouse models and in patients with genetic variants in *Gch1*, independent of NOS expression (Adlam et al., 2012). These findings demonstrate that BH4 has non‐canonical NOS‐independent effects.

Accordingly, we sought to test the requirement for endogenous cardiomyocyte BH4 synthesis in mice with targeted cardiomyocyte deletion of *Gch1*, and to investigate how loss of cardiomyocyte BH4 would alter the functional and metabolic response to cardiac IR injury.

## 2 METHODS

### 2.1 Ethical approval and animal care

The generation and phenotyping of *cmGch1* KO model was carried out in accordance with the Animal (Scientific Procedures) Act 1986, with procedures reviewed by the clinical medicine animal care and ethical review body (AWERB). This study is conducted under project licenses PPL 30/3080 and P0C27F69A at the University of Oxford. Animals were housed in individually ventilated cages (between 4 and 6 mice per cage of mixed genotypes) in specific pathogen‐free conditions. All animals were provided with standard chow (B&K Ltd, Inotiv Mucedola, Milanese, Italy) and water ad libitum and maintained on a 12 h light–12 h dark cycle at controlled temperature (20–22°C) and humidity.

### 2.2 Generation of mice with cardiomyocyte‐specific deletion of *Gch1*

*Gch1^flox/flox^* mice were generated by inserting LoxP sites at either side of exons 2 and 3, which encode the active site of GTPCH, as previously described (Chuaiphichai et al., 2014). Cardiomyocyte‐specific excision of *Gch1* was achieved by crossing *Gch1^flox/flox^* mice with mice expressing heterozygous of α‐myosin heavy chain (α‐MHC)‐driven Cre‐recombinase. Animals expressing *Gch1^floxlflox^* were denoted as WT, and mice with *Gch1^flox/flox^* α‐MHC‐Cre^+/−^ were denoted as cm*Gch1* KO. Littermate controls were used throughout the study. In addition, a batch of *Gch1* ^+/+^ α‐MHC Cre^+/−^ were generated to test the possibility of off‐target effect driven by the Cre recombinase activity. See Supporting information, ‘Supplementary methods’ for details of detecting *Gch1* excision allele and genotyping of the mice. A list of PCR primers is shown in Appendix, Table A1.

### 2.3 Real‐time qPCR analysis of *Gch1* mRNA expression

To confirm the selective deletion of *Gch1* from cardiomyocytes, qRT‐PCR analyses were carried out in isolated adult cardiomyocytes, heart tissue, skeletal muscle and other organs from WT and cm*Gch1* KO mice. β‐Actin was used as an internal control for each sample and the relative quantification of *Gch1* expression between WT and cm*Gch1* KO were determined by the $2^{-\Delta \Delta C_{T}}$ method, where ΔΔ*C*_T_ is the relative fold change to WT. See Supporting information, ‘Supplementary methods’ for detailed protocol.

### 2.4 Measurement of biopterin species

BH4 and oxidised biopterins (BH2 and biopterin) were determined by high‐performance liquid chromatography (HPLC) followed by electrochemical and fluorescence detection, respectively, following an established protocol (Crabtree et al., 2009). Briefly, mice were culled by a Schedule 1 method and the hearts were immediately snap‐frozen in liquid nitrogen. The frozen heart samples were homogenised in 500 μl ice‐cold resuspension buffer (50 mM phosphate‐buffered saline, 1 mM dithiothreitol, 1 mM EDTA, pH 7.4). For isolated adult cardiomyocytes, cells were freeze‐thawed in ice‐cold resuspension buffer three times to break the cell membrane. After centrifugation at 16,000 *g* for 10 min at 4°C, supernatant was removed and ice‐cold acid precipitation buffer (1 M phosphoric acid, 2 M trichloroacetic acid, 1 mM dithioerythritol) was added. Following centrifugation at 16,000 *g* for 10 min at 4°C, the supernatant was removed and injected onto the HPLC system (Jasco, Heckmondwike, UK). Quantification of BH4 and oxidised biopterins was obtained by comparison with external standards and normalised to protein concentration, determined by the bicinchoninic acid protein assay.

### 2.5 Transcriptomic analysis of left ventricular tissue (RNA sequencing)

Total RNA was extracted from the left ventricular tissue of 16‐week‐old WT and cm*Gch1* KO mice (see Supplementary methods in appendix for detail). Extracted RNA was quantified using a NanoDrop spectrophotometer (Thermo fisher Scientific, Carlsbad, USA) and adjusted to 400 ng in 30 μl of ultrapure water; 100 ng of total RNA was used for library preparation. Second strand cDNA synthesis was incorporated with dUTP. The cDNA was end‐paired, A‐tailed and adapter‐ligated. Prior to amplification, samples underwent uridine digestion. The prepared libraries were size selected, multiplexed and quality checked before paired‐end sequencing over two units of a flow cell. Sequencing was performed on a NovaSeq6000 system (Illumina Inc. San Diego, CA, USA) and read counts generated using the tools HISAT2, Picard and feature Counts.

Differential expression analysis by genotype was performed using EdgeR (v 3.28.1) from Bioconductor (Reilingen, Germany). Genes that did not meet the criteria of counts per million (cpm) >3 and a false discovery rate (FDR) <0.05 were excluded from further analysis. The list of differentially expressed genes were then subjected to pathway enrichment analysis by two independent platforms: Ingenuity Pathway Analysis (IPA; Qiagen, Manchester UK) and Cytoscape (v. 3.7.2; Institute for Systems Biology, Seattle, WA, USA) with ClueGo (v. 2.5.6) plugin. Significantly enriched terms (*P* < 0.05) containing a minimum of three genes covering 4% of genes in that term were used to create ClueGo Layouts, where each node represents a GO term. Terms were grouped into networks based on a kappa score level of 0.4.

### 2.6 Western blotting

Frozen left ventricular tissue from 16‐week‐old WT and cm*Gch1* KO mice were homogenised in ice‐cold CelLytic M buffer (Merck Life Science UK Ltd, Gillingham, UK) containing protease and phosphorylase inhibitor cocktails (Roche Applied Science, Mannheim, Germany). The lysates were centrifuged for 5 min at 4°C, and the supernatant were kept for bicinchoninic acid protein assay; 4 μg/μl of samples was prepared using NuPAGE LDS (lithium dodecyl sulfate) Sample Buffer (Thermo Fisher Scientific, Carlsbad, USA) (25%) and reducing agent (10%) (Thermo Fisher Scientific, Carlsbad, USA). Western blotting was carried out using standard protocol with NuPAGE (4–12%, Bis‐Tris) gel and 20 μg of protein were loaded. The working concentration of the primary antibodies were 1:1000, acyl‐coenzyme A dehydrogenase (short‐chain) (Abcam, Cambridge, UK, ab154823), hydroxylacyl‐Coenzyme A dehydrogenase (Abcam ab154088) and long‐chain acyl‐Coenzyme A synthetase (Cell Signalling Technology, MA, USA, 9189S). Appropriate fluorescent secondary antibodies (Li‐Cor Biosciences) were applied, and the membrane were imaged using the Li‐Cor Odessy system. For immunodetection of nNOS (Santa Cruz Biotechnology, Dallas, TX, USA, sc‐5302), horseradish peroxidase‐conjugated secondary antibodies (Promega, Madison, WI, USA) was used.

### 2.7 Triacylglycerol assay

Assessment of intracellular triacylglycerol (TAG) from left ventricular tissue of the mice was measured spectrophotometrically using Randox TAG kit (TR210, Randox Laboratories, Crumlin, UK) following Folch TAG extraction. Briefly, 500 μl of Folch (2:1 chloroform:methanol) was added to 10 mg of tissue and homogenised with a Precellys homogeniser for 30 s at 5500 rpm. The samples were then centrifuged at 5000 *g* at room temperature for 15 min. The lipid phase was transferred into a new Eppendorf tube and evaporated with lid open at 45–50°C for 1.5 h. Two hundred microlitres of ethanol was used to resuspend the samples and left to evaporate overnight at room temperature. The next day, the samples were resuspended in 20 μl of ethanol and then a TAG assay was carried out according to the manufacturer's instructions.

### 2.8 In vivo assessments of cardiac function

In vivo cardiac function assessments were obtained by echocardiography (Vevo 2200, FujiFilm Sonosite Inc, Bedford, UK) at an age of 16 weeks. Mice were anaesthetised (1.5–2% isoflurane in O_2_), body temperature was maintained at 37°C, and images were taken at a heart rate of 480–510 bpm. Cardiac dimension and function were assessed by M‐mode echocardiography. Left posterior wall thickness and left ventricular internal diameter at end‐systole and end‐diastole were measured and values were averaged from three heartbeats. Ejection fraction and fractional shortening were derived from the heart dimensions with in‐built equations of the Vevo2200 system.

### 2.9 ECG recording and analysis

Surface ECGs were obtained in anaesthetised mice (2% isoflurane, 0.5 l/min O_2_) by placing platinum subdermal needle electrodes in each limb, which was connected to an Iso‐DAM8A amplifier (World Precision Instruments, Sarasota, FL, USA) and a CED Power 1401‐3A interface (Cambridge Electronic Design Ltd, Cambridge, UK). ECG data were acquired using Spike2 electrophysiology software (Cambridge Electronic Design). Mice were stabilised for 5 min and the recordings from this period were not included in the analysis. After the stabilisation period, 2‐min consecutive ECG traces were used to form an averaged waveform and the standard ECG parameters were measured: P wave duration, P–Q interval, QRS duration, corrected QT interval (QTc) and R–R interval. QTc values were calculated as per Bazett's formula, modified for mice: $\mathrm{QTc}\;=\;\mathrm{QT}/\surd (\frac{\mathrm{RR}}{100})$ (Mitchell et al., 1998).

### 2.10 *Ex vivo* ischaemia–reperfusion injury model

*Ex vivo* IR injury of the mouse heart was assessed using a Langendorff system as previously described (Moyes et al., 2019). Animals were terminally anaesthetised with intraperitoneal injection of a mixture of heparin (300 U), ketamine (75 mg/kg) and medetomidine (1 mg/kg). Hearts were quickly excised and placed into ice‐cold Krebs–Henseleit (KH) buffer (composition: NaCl 118.5 mM; KCl 4.7 mM; MgSO_4_ 2.4 mM; KH_2_PO_4_ 1.2 mM; glucose 11.9 mM; NaHCO_3_ 25.0 mM; C_3_H_3_NaO_3_ 2.0 mM and CaCl_2_ 1.7 mM; pH 7.4; gassed with 95% O_2_–5% CO_2_). Hearts were retrogradely perfused with KH buffer at constant flow of 2 ml/min, at 37°C. The left atrium was then removed, and an intraventricular balloon inserted into the left ventricle (LV) for measurements of LV developed pressure (LVDP), which was calculated by the difference between systolic and diastolic pressure; heart rate was derived from LV contractions per minute. Hearts were stabilised for 20 min and then global cessation of flow was induced for 35 min, followed by 60 min of reperfusion. Hearts with heart rate less than 280 bpm and LVDP smaller than 70 mmHg after the stabilisation period were excluded.

### 2.11 Triphenyl tetrazolium chloride staining

Following *ex vivo* IR, hearts were removed from the Langendorff and briefly frozen at −20°C then sliced into 1 mm‐thick sections on the transverse axis of the heart. The heart slices were then incubated with 1% triphenyl tetrazolium chloride (TTC) in phosphate‐buffered saline at 37°C for 15 min. After the incubation, excess fluid was removed from the heart sections and then photographed with a scanner on both sides. Infarcted area (white) was measured using ImageJ and expressed as a percentage of total ventricular area. The final infarcted size was averaged from both side of the scanned image.

### 2.12 Metabolomics

To investigate the metabolic changes in response to IR injury, a set of hearts from female WT and cm*Gch1* KO mice were subjected to one of the four time points on the Langendorff mode as described above: (1) sham (75 min perfusion), (2) 35 min ischaemia followed by 30 min reperfusion, (3) 35 min ischaemia plus 5 min reperfusion, and (4) 35 min ischaemia. Whole hearts were immediately snap‐frozen in liquid nitrogen and stored at −80°C; 70–100 mg of the frozen tissue sample were shipped to Metabolon (Durham, NC, USA) on dry ice. Metabolon measured metabolites using ultrahigh performance liquid chromatography–tandem mass spectroscopy (UPLC‐MS/MS). Details of sample preparation and the protocol for UPLC‐MS/MS used by Metabolon has been previously described (Bailey et al., 2019).

### 2.13 Data and statistical analysis

All data are reported as means ± SD. The experimental unit (*n*) was defined as a single animal. Animals of both genotypes were caged together, and animals of both genotypes were derived from more than one cage in all experiments. Age‐ (16 weeks old) and sex‐matched mice were randomly assigned to experiments. Both sexes were used throughout the study unless stated otherwise. Statistical analyses were done using GraphPad Prism version 9.3.0 (GraphPad Software Inc., San Diego, CA, USA). A two‐tailed, unpaired Student's *t*‐test was used to compare two groups affected by one single variable. In the cases of comparing more than two groups affected by one variable, multiple unpaired *t*‐tests were used. Two‐ways ANOVA was used to compare data influenced by two factors, with the Bonferroni *post hoc* test. For all comparisons, *P* < 0.05 was considered statistically significant. RNA‐seq dataset are available on the Gene Expression Omnibus data repository: database accession number: GSE206063.

## 3 RESULTS

### 3.1 Development and characterisation of cm*GCh1* KO mice

**FIGURE 1:**
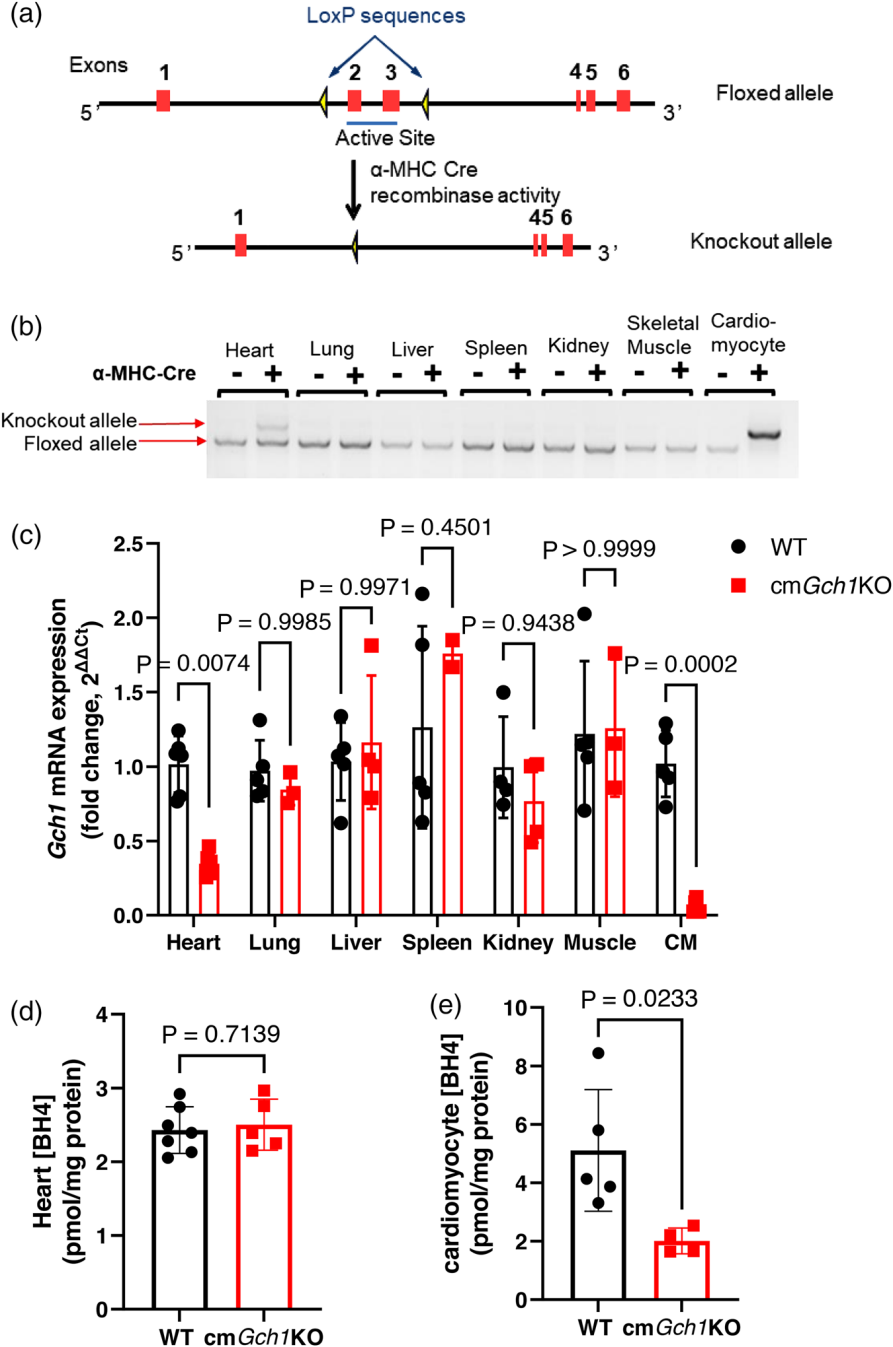
Generation and characterisation of cardiomyocyte‐specific deletion of *Gch1*. (a) Schematic diagram of LoxP site insertion and the resultant excised allele after crossing with α‐MHC Cre line. (b) A representative gel image of genomic DNA PCR products showing the knockout allele and floxed allele in tissue from mice with (+) or without (−) α‐MHC Cre across different tissue types. (c) *Gch1* mRNA expression in different tissues and isolated cardiomyocytes (CM). (d,e) BH4 levels in the heart (d) and in cardiomyocytes (e). Data are presented as means ± SD and analysed using multiple *t*‐tests (c) or an unpaired Student's *t*‐test (d,e). Each data point represents an individual animal.

**FIGURE A1:**
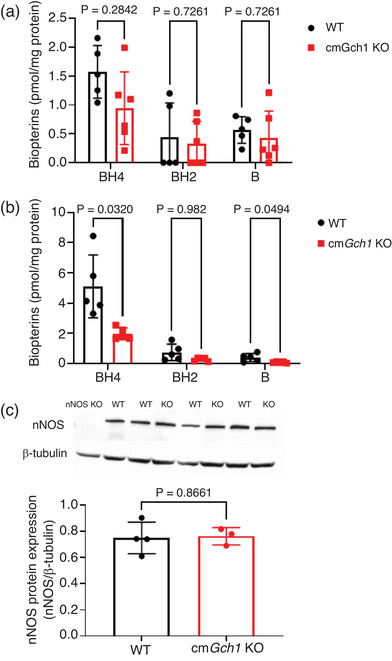
Measurements of biopterin species using HPLC in isolated adult cardiac endothelial cells and cardiomyocytes. (a) Levels of biopterin species in isolated adult cardiac endothelial cells. (b) Levels of biopterin species in isolated adult cardiomyocytes. (c) nNOS protein expression in the heart. Data are presented as means ± SD and analysed with multiple unpaired *t*‐tests (a, b) and single unpaired *t*‐test (c).

We investigated the requirement for BH4 synthesis in cardiomyocytes by targeted deletion of *Gch1*, encoding GTPCH, the enzyme responsible for *de novo* BH4 synthesis (Figure 1a). As shown in Figure 1b, excision of the floxed *Gch1* allele was observed only in cardiomyocytes from cm*Gch1* KO mice, with almost complete abolition of *Gch1* mRNA expression, compared to WT cardiomyocytes (Figure 1c, *P* = 0.0002). In contrast, *Gch1* mRNA expression in whole heart tissue from cm*Gch1* KO hearts was reduced by only ∼60% compared with WT (*P* = 0.0074), indicating that *Gch1* is expressed in other cardiac cells such as endothelial cells and cardiac fibroblasts. Furthermore, there were no differences between WT and cm*Gch1* KO mice in *Gch1* mRNA expression across other tissue types, confirming the specificity of *Gch1* deletion in cardiomyocytes. Moreover, BH4 levels were significantly reduced in *Gch1* KO cardiomyocyte, compared to WT (*P* = 0.0233), but not in the whole heart (*P* = 0.7139) (Figure 1d,e). This observation indicates that BH4 is produced by other cell types in the heart, such as endothelial cells, at a greater level than in cardiomyocytes (Chuaiphichai et al., 2014; Chuaiphichai et al., 2017). Correspondingly, BH4 levels in isolated cardiac endothelial cells between cm*Gch1* KO and WT mice were comparable (*P* = 0.2842) (Appendix, Figure A1a). In addition, total levels of biopterin were reduced in cm*Gch1* KO cardiomyocytes but not the levels of BH2 (Appendix, Figure A1b), suggesting that cardiomyocyte BH2 may be taken up from plasma or other surrounding cells. Furthermore, expression of nNOS, the most abundant NOS subtype in cardiomyocyte, was not altered by BH4 availability (Appendix, Figure A1c).

### 3.2 Transcriptomics reveals substrate metabolic pathway changes in hearts with cardiomyocyte‐specific deletion of *GCh1*

**FIGURE 2:**
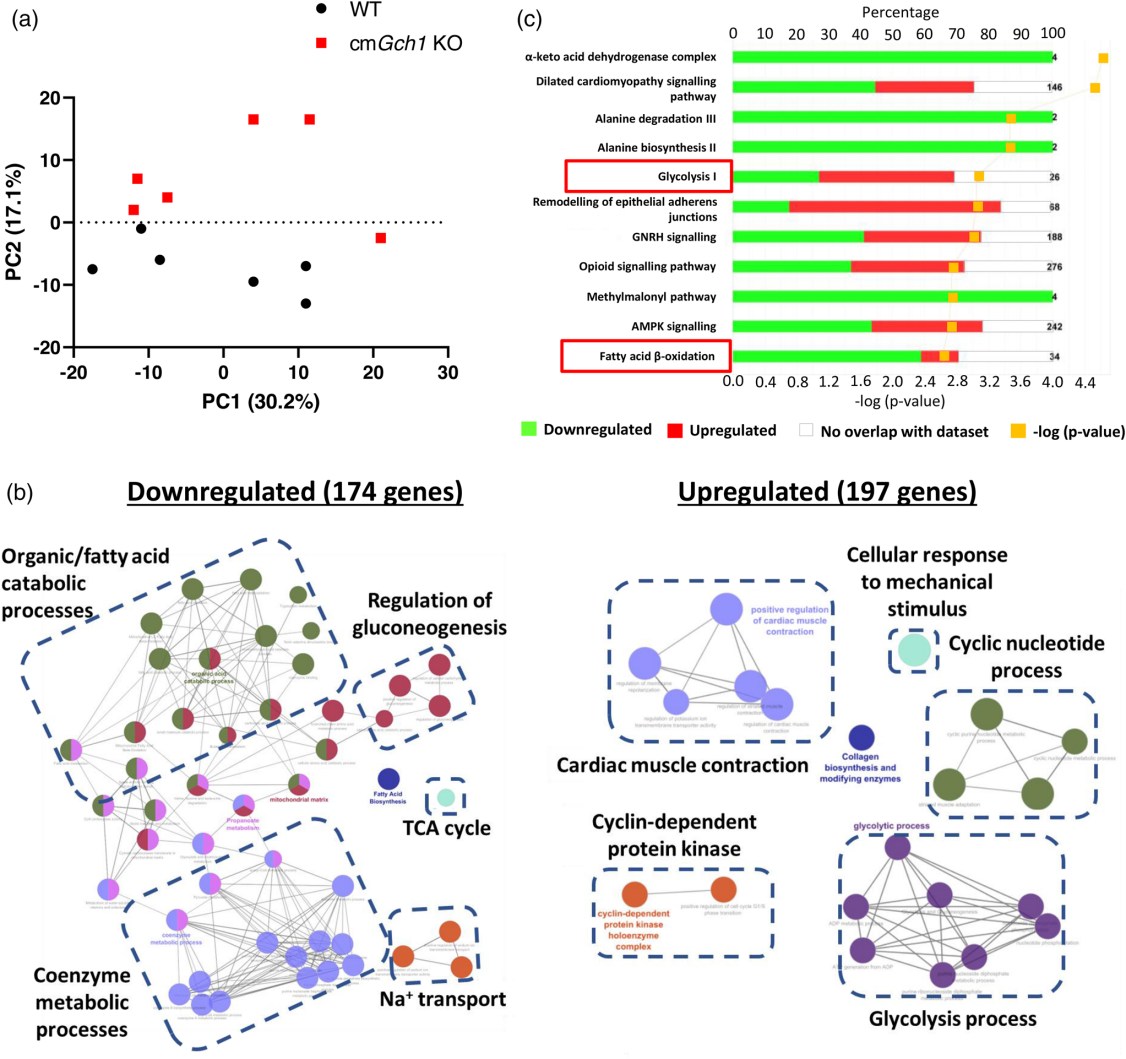
RNA sequencing of hearts from WT and cm*GCh1* KO mice reveals downregulation of fatty acid metabolism genes. (a) Principal component (PC) analysis plot of RNA‐seq data of WT and cm*Gch1* KO hearts. (b) Cytoscape layout of pathway analysis using ClueGo plug‐in; each node represents a GO term and they are connected if 4% of genes overlapped. (c) Enrichment pathway analysis of the RNA‐seq data using IPA software. Percentages of downregulated and upregulated genes over the total known genes associated with the pathways are shown in green and red, respectively*. P‐*value of the pathway significance is shown by yellow squares*. n* = 6, genes with FDR < 0.05 are considered significantly differentially expressed and used in pathway analysis. TCA, tricarboxylic acid.

**FIGURE A2:**
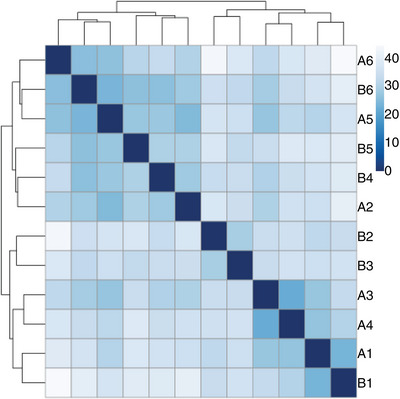
Hierarchical clustering heatmap. Hierarchical clustering was performed on full transcriptomic data using Bioconductor R. Sample A1–6 were from WT mice and sample B1–6 were from cm*Gch1* KO mice.

We first sought to investigate whether any gene expression alterations in cm*Gch1* KO mice could have an impact on cardiac metabolism and function. Therefore, we undertook transcriptome analyses of cm*Gch1* KO and WT hearts by RNA sequencing. We found 174 genes that were downregulated and 197 genes that were upregulated in cm*GCh1* KO hearts compared with WT. A principal component analysis (PCA) captured 47.3% of the transcriptome data, and the data were plotted based on the similarities of the sample gene expression (Figure 2a). Hierarchical clustering was then performed based on the whole transcriptomic profiles and showed no clear clustering of WT versus cm*Gch1* KO samples (Appendix, Figure A2). Although there was no distinctive clustering between the genotypes, pathway enrichment analysis using Cytoscape ClueGo revealed downregulation of genes involved in fatty acid metabolism in cm*Gch1* KO hearts, whilst genes related to glycolysis were upregulated (Figure 2b). Genes associated with the ClueGo term ‘fatty acid metabolism’ are listed in Appendix, Table A2. In addition, regulation of gluconeogenesis, tricarboxylic acid cycle, coenzyme metabolic processes and sodium membrane transport were downregulated, whereas pathways associated with cardiac muscle contraction, cellular response to mechanical stimulus, cyclic nucleotide process and cyclin‐dependent protein kinase were upregulated.

Similarly, IPA pathway analysis also revealed enrichment of genes involved in glycolysis and fatty acid metabolism (Figure 2c). Taken together, both the Cytoscape ClueGo platform and IPA pathway analysis independently identified a change in energy metabolism, favouring a metabolic shift within the myocardium towards glycolysis and away from fatty acid oxidation in cm*Gch1* KO hearts.

### 3.3 Enzymes involved in fatty acid catabolism are downregulated in cardiomyocyte‐specific deletion of *Gch1*

**FIGURE 3:**
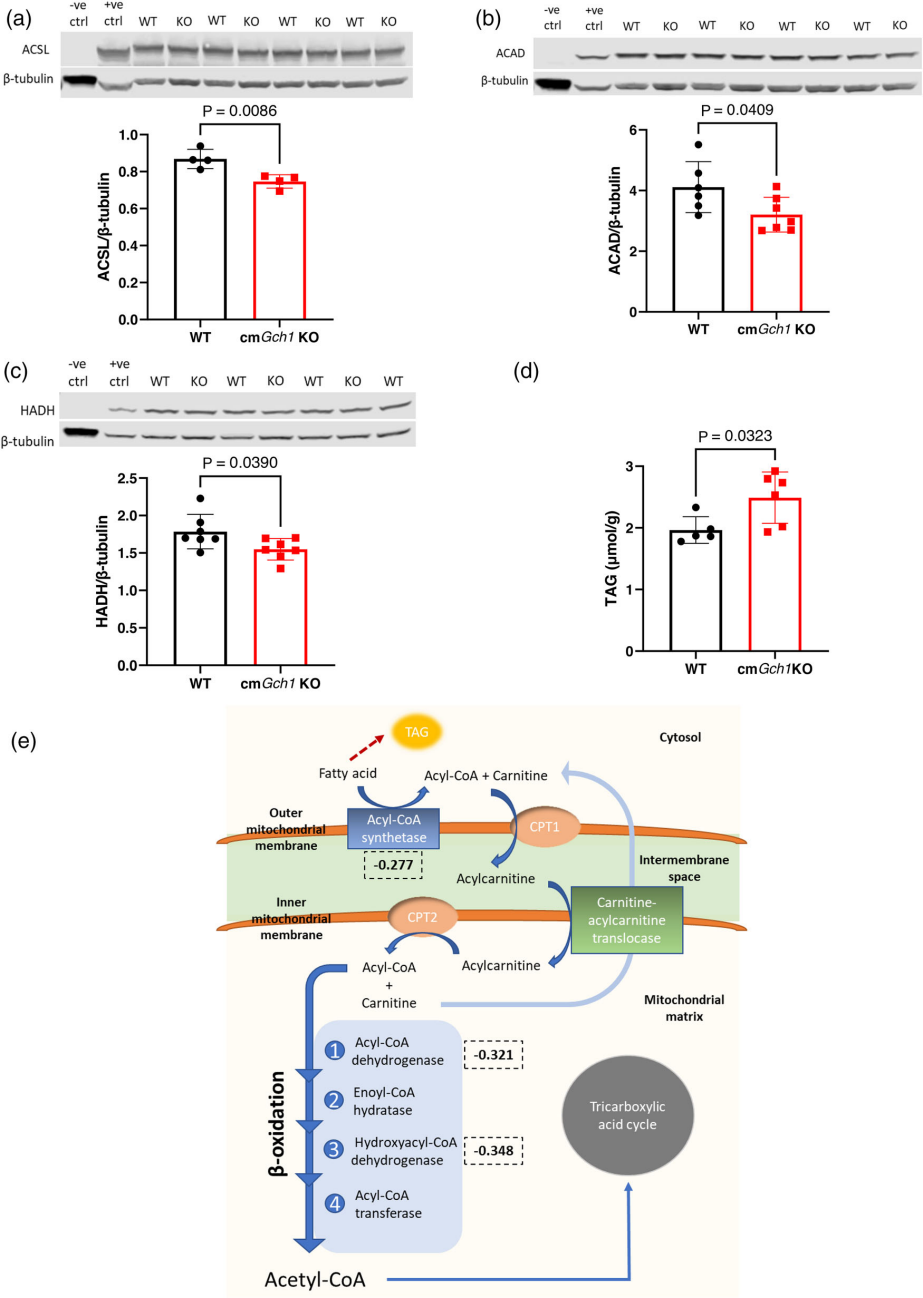
Downregulation of enzymes involved in fatty acid oxidation in cm*Gch1* KO hearts. (a–c) Protein levels of three key fatty acid oxidation enzymes (ACSL, acyl‐coenzyme A synthase (long‐chain); ACAD, acyl‐coenzyme A dehydrogenase; HADH, hydroxylacyl‐coenzyme A dehydrogenase). Brain and liver tissues were used as negative (−ve) and positive (+ve) controls, respectively. (d) Intracellular triacylglycerol (TAG) levels are increased in cm*Gch1* KO hearts compared to WT. (e) A schematic diagram of fatty acid catabolism pathway. Fatty acid can either undergo oxidation for ATP production or be incorporated into TAG. Fold changes (log) of mRNA expression by RNA sequencing are shown in dotted boxes. Data are presented as means ± SD and analysed using unpaired Student's *t*‐test. Each data point represents an animal used.

**FIGURE A3:**
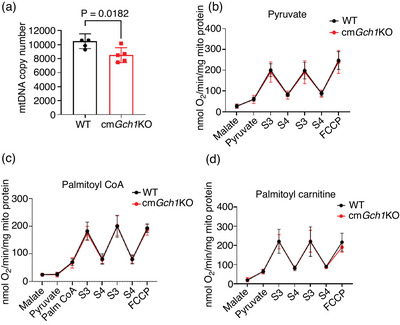
Mitochondrial DNA copy number and cardiac mitochondrial oxidative phosphorylation rate. (a) Cardiac mitochondria (mt) DNA copy number averaged from 2^Δ^ *^C^* ^T^ of *Actb* versus *Cytb*, and *sdha* versus *mtCo2*. (b–d) Oxidative phosphorylation in isolated mitochondria from WT and cm*Gch1* KO hearts using Clark electrode. Data are presented as means ± SD and analysed using Student's *t*‐test (a) and two‐way ANOVA (b–d). *n* = 5.

In keeping with downregulated fatty acid metabolism discovered in pathway enrichment analysis, we next identified that protein levels of three key enzymes involved in fatty acid oxidation (acyl‐CoA synthetase, acyl‐CoA dehydrogenase and hydroxyacyl‐CoA dehydrogenase) were decreased in cm*Gch1* KO hearts (*P* = 0.0086, *P* = 0.0409 and *P* = 0.0390, respectively) (Figure 3a–c). Fatty acids imported into the heart can either be oxidised in the mitochondria or esterified into TAG. The decrease in fatty acid oxidation enzymes was accompanied by a significant increase in myocardial TAG concentration in cm*Gch1* KO cardiac tissue compared to WT (*P* = 0.0323) (Figure 3d), demonstrating a diversion of fat away from catabolic and into anabolic pathways. A schematic diagram of fatty acid processes is illustrated in Figure 3e. Fatty acid oxidation takes place in mitochondria. Mitochondrial DNA copy number was lower in cm*Gch1* KO compared to WT hearts (*P* = 0.0182) (Appendix, Figure A3), consistent with the downregulation of fatty acid oxidation. In line with this, mitochondrial oxidative phosphorylation using the glycolysis product pyruvate, fatty acid derivatives, palmitoyl coenzyme A and palmitoyl carnitine showed no differences in oxygen consumption rate in isolated mitochondria from WT and cm*Gch1* KO hearts (Appendix, Figure A3), demonstrating that the activity of enzymes involved in cardiomyocyte oxidative phosphorylation is not altered by BH4 deficiency.

### 3.4 Deletion of cardiomyocyte *Gch1* has no effect on basal cardiac function

**FIGURE A4:**
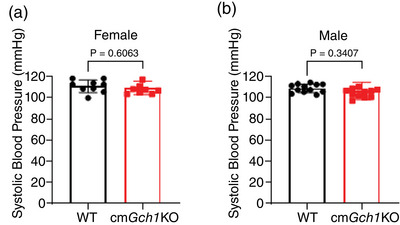
Blood pressure measurement at 16 weeks old for WT and cm*Gch1* KO mice. Data are presented as means ± SD and analysed with Student's *t*‐test.

We next investigated whether the metabolic changes observed in the hearts of cm*Gch1* KO mice impacted on cardiac function. We did not observe significant changes in echocardiographic parameters of cardiac dimensions or function between cm*Gch1* KO and WT, in either female or male animals (Table 1), nor were there any differences in heart rate or ECG characteristics (Table 2). Loss of *Gch1* expression in cardiomyocytes did not affect blood pressure (Appendix, Figure A4).

**TABLE 1:** Basal cardiac dimensions and function.

Baseline cardiac function	WT	cm*Gch1* KO	*P*
Female			
LVID;d (mm)	3.40 ± 0.17	3.40 ± 0.25	0.968
LVID;s (mm)	1.95 ± 0.21	2.02 ± 0.22	0.494
LVPW;d (mm)	0.64 ± 0.13	0.68 ± 0.12	0.503
LVPW;s (mm)	1.16 ± 0.08	1.17 ± 0.13	0.906
EF (%)	74.80 ± 4.74	72.64 ± 3.84	0.304
FS (%)	42.81 ± 4.35	40.83 ± 3.28	0.292
Male			
LVID;d (mm)	3.65 ± 0.16	3.71 ± 0.30	0.5
LVID;s (mm)	2.04 ± 0.22	2.14 ± 0.39	0.451
LVPW;d (mm)	0.82 ± 0.11	0.84 ± 0.13	0.709
LVPW;s (mm)	1.36 ± 0.10	1.35 ± 0.09	0.788
EF (%)	75.97 ± 4.71	74.16 ± 7.25	0.454
FS (%)	44.10 ± 4.28	42.81 ± 6.55	0.553

Unconscious echocardiography measurements were taken from 16‐week‐old mice. Data are presented as mean ± SD and analysed using Student's *t*‐test for comparison of genotype in each parameters. WT, female *n* = 9, male *n* = 13; cm*Gch1* KO, female *n* = 9, male *n* = 14. LVID, left ventricular internal diameter; LVPW, left ventricular posterior wall; s, at end‐systole; d, at end‐diastole; EF, ejection fraction; FS, fractional shortening.

**TABLE 2:** Baseline ECG parameter.

Baseline ECG (ms)	WT	cm*Gch1* KO	*P*
Female			
P wave duration	14.40 ± 1.05	14.41 ± 1.24	0.985
P–Q interval	42.38 ± 2.15	40.70 ± 4.25	0.409
QRS duration	10.44 ± 1.07	11.05 ± 0.37	0.22
QTc	48.73 ± 6.90	46.43 ± 4.72	0.516
R–R interval	120.99 ± 6.29	125.11 ± 11.52	0.459
Heart rate (bpm)	485.0 ± 23	495 ± 39	0.591
Male			
P wave duration	13.95 ± 1.88	14.25 ± 0.91	0.63
P–Q interval	36.18 ± 2.38	37.96 ± 2.66	0.116
QRS duration	10.16 ± 0.70	10.76 ± 0.67	0.054
QTc	55.44 ± 4.81	52.53 ± 7.22	0.289
R–R interval	117.21 ± 15.96	123.80 ± 12.13	0.284
Heart rate (bpm)	519 ± 64	489 ± 50	0.229

Unconscious ECGs were taken from 16 weeks old WT and cm*Gch1* KO mice. Data are presented as means ± SD and analysed using Student's *t*‐test for comparison of genotype in each parameters. WT, female *n* = 6, male *n* = 10; cm*Gch1* KO, female *n* = 6, male *n* = 12.

### 3.5 Cardiomyocyte‐specific deletion of *Gch1* protects against ischaemia–reperfusion injury

**FIGURE 4:**
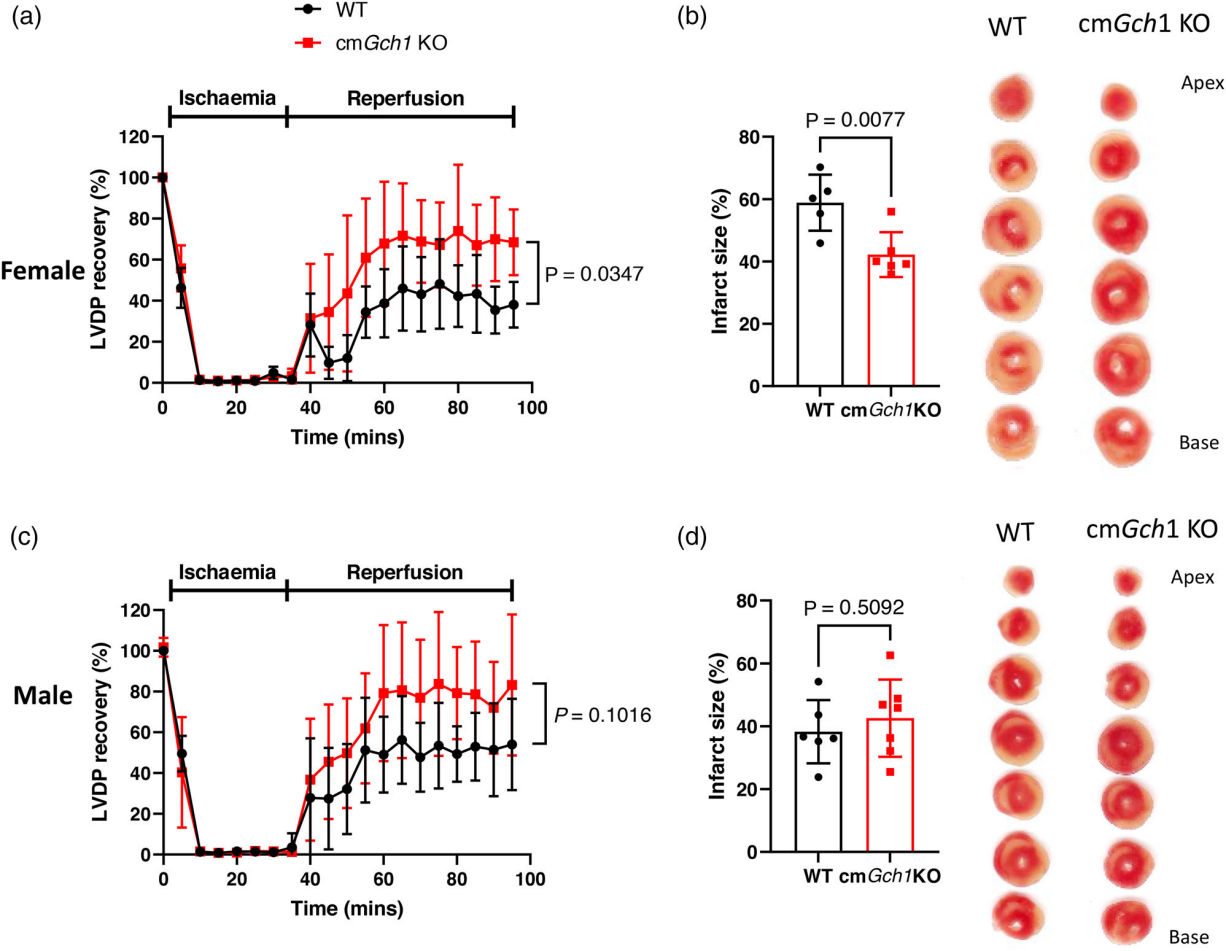
Genetic deletion of *Gch1* in cardiomyocyte protects against ischaemia–reperfusion injury. Recovery of left ventricular development pressure (LVDP) and final infarct size in female (a,b) and male (c,d) from WT and cm*Gch1* KO mice subjected to 35 min global ischaemia (zero flow) followed by 60 min reperfusion. Representative scanned images of infarcted heart sections are shown. Data are presented as means ± SD and analysed using two‐way ANOVA with Bonferroni *post hoc* test (a,c) or unpaired Student's *t*‐test (b,d). Each data point represents an individual animal (*n* = 5–7 per group).

**FIGURE A5:**
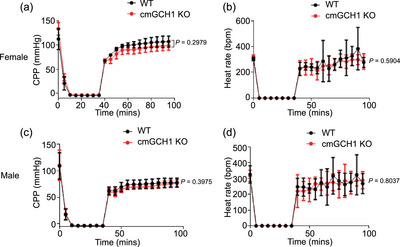
Coronary perfusion pressure (CPP) and heart rate of the isolated Langendorff heart model in male and female mice. The hearts were subjected to 35 min global ischaemia (zero flow) followed by 60 min reperfusion (2 ml/min constant flow). Data are presented as means ± SD and analysed using two‐way ANOVA.

**FIGURE A6:**
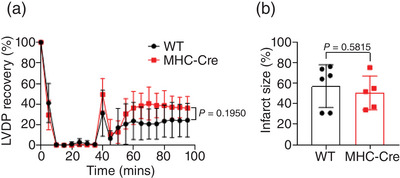
Functional recovery of the left ventricular and infarct size does not affect by α‐MHC Cre activity. (a) Recovery of left ventricular development pressure (LVDP) and (b) the final infarct size in isolated Langendorff hearts from female WT and *Gch1^+/+^* α‐MHC Cre^+/−^ mice subjected to 35 min global ischaemia (zero flow) followed by 60 min reperfusion (2 ml/min constant flow). Data are presented as means ± SD and analysed using two‐way ANOVA (a) or Student's *t*‐test (b).

Since cardiomyocyte‐specific loss of BH4 did not impact on basal cardiac function, we next used the Langendorff isolated heart model to test whether cardiomyocyte BH4 deficiency would alter cardiac function in response to cardiac injury. Deletion of *Gch1* in cardiomyocytes of cm*Gch1* KO mice protected against IR injury as demonstrated by improved recovery of left ventricular function (*P* = 0.0347) (Figure 4a). In female cm*Gch1* KO mice, there was a significant reduction in hyper‐contracture at the initial phase of reperfusion compared to WT, and thereafter left ventricular function was persistently superior to WT, suggesting a protective mechanism that arises during the early phase of reperfusion. The greater recovery in left ventricular function was associated with a significantly reduced infarct size (WT, 58.85 ± 9.02%; cm*Gch*1 KO 42.19 ± 7.19%; *P* = 0.0077). However, the LVDP recovery in male between WT and cm*Gch1* KO did not reach statistical significance (*P* = 0.1016) and there were no differences in the final infarct size (*P* = 0.5092) (Figure 4c,d). In addition, there was no difference in coronary perfusion pressure and heart rate between the genotypes (Appendix, Figure A5). To assure that the phenotype observed were not due to off‐target effects of α‐MHC Cre activity, we subjected *Gch1^+/+^* α‐MHC Cre^+/−^ control hearts to the IR injury protocol. We observed similar LVDP recovery patterns between *Gch1^+/+^* α‐MHC Cre^+/−^ and WT hearts and no difference in infarct size (*P* = 0.1950 and *P* = 0.5815) (Appendix, Figure A6), indicating that the protective phenotype only occurs in cm*Gch1* KO hearts.

### 3.6 Post‐ischaemic metabolomic profiles

**FIGURE 5:**
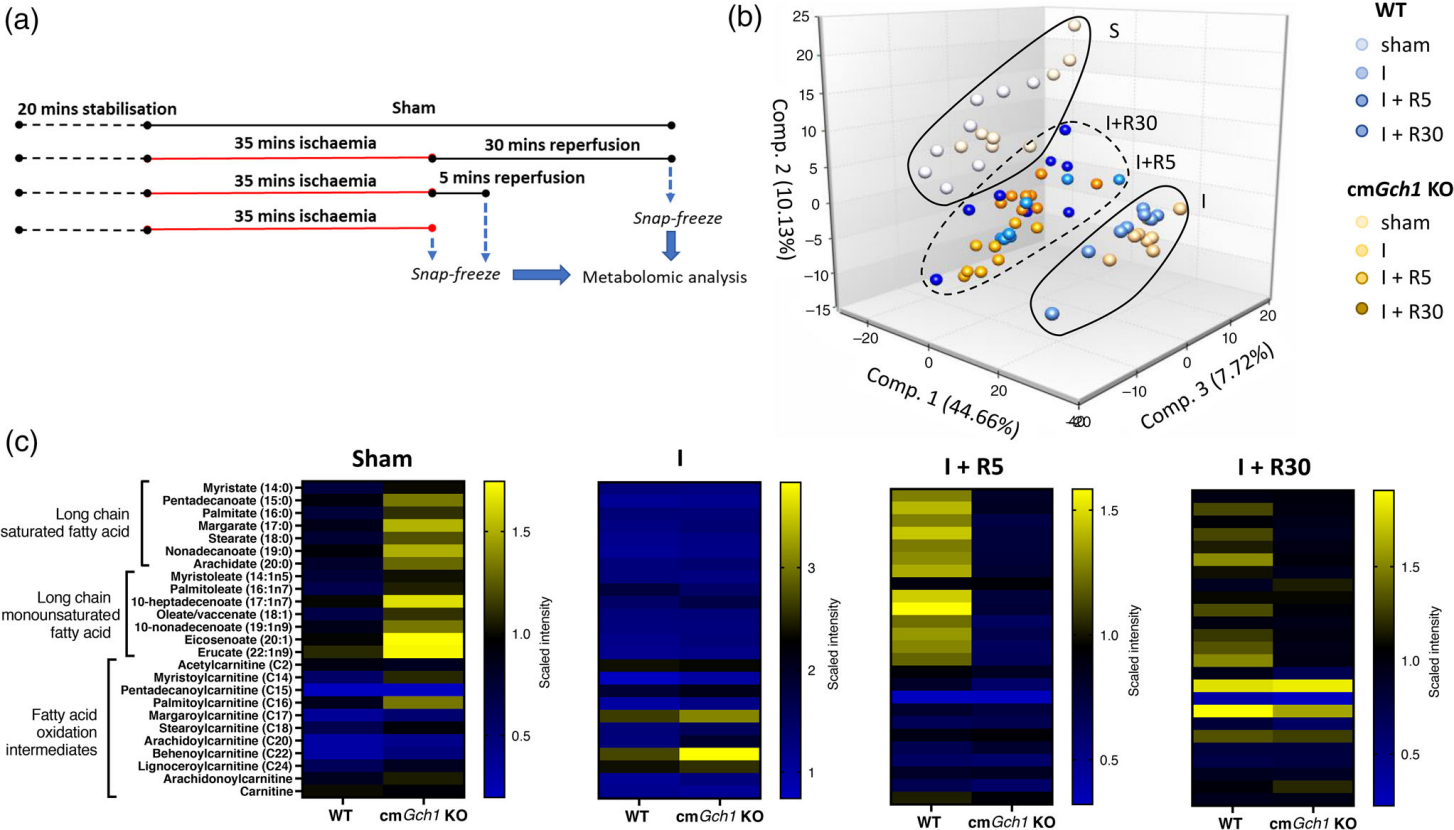
Metabolomic analysis of long‐chain fatty acid and its oxidation intermediates from WT and cm*Gch1* KO hearts in response to *ex vivo* ischaemia–reperfusion (IR) injury. (a) A diagram showing the sample collection protocol. Langendorff isolated hearts were subjected to sham, 35 min global ischaemia (I), or 35 min global ischaemia followed by either 5 min reperfusion (I + R5) or 30 min reperfusion (I + R30). Snap‐frozen samples were subject to metabolomic analysis. (b) Principal component analysis plot of the metabolomic data from the IR injury hearts. Shapes are drawn around the data points for visualisation. (c) Heatmaps showing levels of long‐chain fatty acids and their oxidation intermediates at each time point of IR. Data are presented as the mean scaled intensity, normalised from the original raw detection intensity. *n* = 7–8 in each group.

**FIGURE A7:**
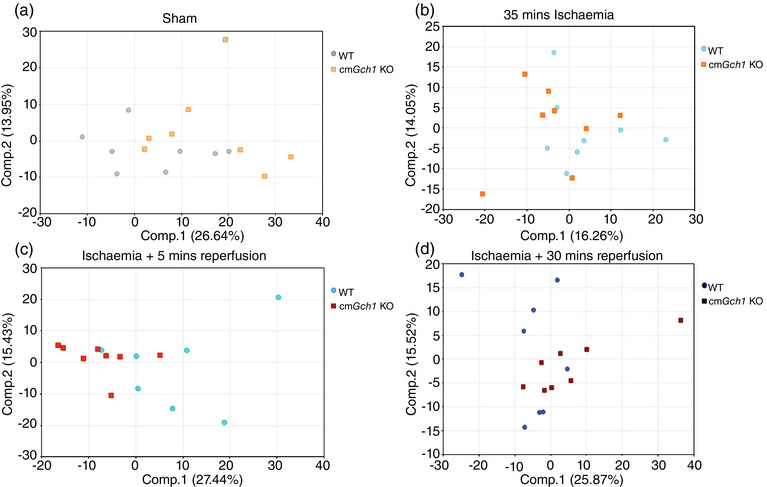
PCA plots of the metabolomic data from the different time points of ischaemia reperfusion injury. *n* = 7–8.

We hypothesised that the protective phenotype observed in cm*Gch1* KO mice in response to IR injury could be due to altered cardiac metabolism, favouring glycolysis with downregulation of fatty acid oxidation. To investigate the effects of cardiomyocyte‐specific deletion of *Gch1* on the metabolic response to IR injury, we undertook a non‐biased metabolomics analysis, using female mice as the effect on IR injury was more prominent. Hearts from WT or cm*Gch1* KO mice were immediately snap‐frozen in liquid nitrogen at one of four time‐points: (1) sham, (2) 35 min ischaemia followed by 30 min reperfusion (I + R30), (3) 35 min ischaemia plus 5 min reperfusion (I + R5), and (4) 35 min ischaemia (I) (Figure 5a). The PCA plot (Figure 5b) showed the sham and ischaemia groups as distinctive clusters, reflecting the marked metabolic changes induced by ischaemia. The ischaemia plus reperfusion groups were clustered between the sham and ischaemia groups, with overlapping of the 5 min and 30 min reperfusion. However, there was no strong separation between the WT and cm*GCh1* KO samples within each condition (Appendix,Figure A7), indicating that the effect of *Gch1* deletion on the cardiomyocyte response to IR is not associated with global changes in cardiac metabolites.

**FIGURE A8:**
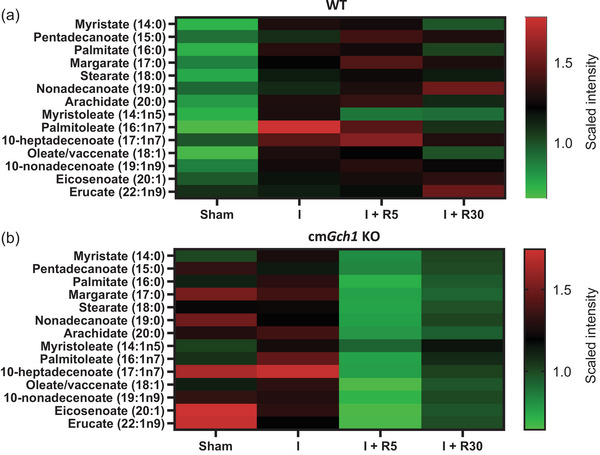
Metabolomic analysis of long‐chain fatty acid metabolites from WT and cm*Gch1* KO hearts in response to *ex vivo* IR injury. (a, b) Heatmaps showing levels of fatty acid metabolites from Langendorff isolated hearts subjected to sham, 35 min ischaemia (I), 35 min ischaemia followed by 5 min reperfusion (I + R5), or 35 min ischaemia followed by 30 min reperfusion (I + R30). Data are presented as the mean scaled intensity that were normalised from the original raw detection intensity. *n* = 7–8 in each group.

Accordingly, we next sought to identify specific groups of metabolites, and pathways that might underlie the differences in the response to IR injury between cm*Gch1* KO and WT hearts. We found that a large group of long‐chain fatty acids increased in cm*Gch1* KO samples compared to WT (Figure 5c). However, the mitochondrial intermediates of fatty acid oxidation, namely the long‐chain and medium‐chain acyl carnitines, were not different between WT and cm*Gch1* KO, demonstrating again that the changes in metabolism in cm*Gch1* KO hearts were occurring at the level of the mitochondria and driving upstream accumulation of fatty acids within the cytosol. In WT mice the levels of long‐chain fatty acids were higher in the groups subject to I, I + R5 and I + R30 compared with sham (Appendix, Figure A8a). In contrast, in cm*Gch1* KO mice the I + R5 group had the lowest levels of long‐chain fatty acids compared with sham and I groups (Appendix, Figure A8b). When comparing the genotypes in each condition (Figure 5c), the levels of fatty acid at I + R5 were higher in WT than cm*Gch1* KO mice, illustrating a key mechanism that could have led to the protective phenotype of the cm*Gch1* KO hearts.

## 4 DISCUSSION

This study identifies and characterises a requirement for cell‐specific synthesis of BH4 in cardiomyocytes, using a novel targeted mouse model of cardiomyocyte deletion of *Gch1*. The principal findings are that, first, loss of *de novo* BH4 synthesis by genetic deletion of *Gch1* within cardiomyocytes results in cardiomyocyte BH4 deficiency, but without complete loss of cardiac BH4 levels, reflecting the contribution of BH4 synthesis in endothelial cells and fibroblasts. Second, cardiomyocyte‐specific *Gch1* deletion is associated with altered myocardial fatty acid metabolism, with reduced levels of fatty acid oxidation enzymes, increased long‐chain fatty acid and TAG levels. Third, the altered energy metabolism in the heart due to cardiomyocyte BH4 deficiency does not affect basal cardiac function, but alters the response to IR injury, with improved functional recovery after IR, and reduced infarct size. These molecular and physiological effects modulated by BH4 availability could possibly be via NOS‐independent pathways as NOS expressions were not altered in *Gch1* KO hearts. Taken together, these findings identify new cell‐specific roles and requirements for *Gch1* and BH4 in cardiomyocyte fatty acid metabolism, and the response to myocardial IR injury.

The cm*Gch1* KO mouse provides a new model to understand the cell‐ and tissue‐specific relationships between *Gch1* expression, BH4 synthesis and BH4 recycling or uptake. *Gch1* mRNA expression was almost completely abolished in isolated cardiomyocytes from cm*Gch1* KO mice, whereas BH4 levels were maintained at approximately 40% of the level seen in WT mice. This is in contrast to other cell‐specific *Gch1* knockout models, such as endothelial cells (Chuaiphichai et al., 2014) or inflammatory cells (McNeill et al., 2015), where *Gch1* deletion results in almost complete loss of BH4 in the targeted cells. This indicates that neither BH4 uptake from plasma nor recycling of BH4 from BH2 via dihydrofolate reductase (Bendall et al., 2014; Hasegawa et al., 2005) is sufficient to rescue cell‐specific BH4 deficiency in endothelial and inflammatory cells with loss of *de novo* BH4 synthesis. In contrast, the fact that BH2 levels were not significantly altered in cm*Gch1* KO cardiomyocytes supports the notion that BH4 recycling may be maintained in cardiomyocytes via the salvage pathway, possibly via uptake of BH4/BH2 from plasma or other myocardial cells. Indeed, the myocardium can be effectively targeted by systemic augmentation of BH4, via dietary supplementation (Moens et al., 2008), whereas endothelial cell BH4 is not increased by oral BH4 supplementation (Chuaiphichai et al., 2021; Cunnington et al., 2012). Nevertheless, the resulting level of BH4 in cm*Gch1* KO cardiomyocytes remains insufficient to rescue the requirement for BH4 in cardiac metabolism, as reflected by the alterations in substrate metabolism pathways. It is possible that localised intracellular production of BH4 is important, especially in relation to mitochondria, where fatty acid metabolism takes place. Indeed, a previous study found that mitochondrial BH4 levels were reduced in response to pressure‐overload causing cardiac hypertrophy and fibrosis, whereas BH4 in the cytosol and microsome was unchanged (Shimizu et al., 2013). This suggests that cardiomyocyte BH4 is compartmentalised, raising the possibility that BH4 availability may be differentially regulated in subcellular compartments by *de novo* synthesis, recycling or uptake.

The changes in fatty acid metabolism resulting from cardiomyocyte *Gch1* deletion were consistent in the mRNA expression and in the protein levels of fatty acid oxidation enzymes, and in the myocardial levels of multiple long‐chain fatty acids and TAG. Previous studies have demonstrated that BH4 is involved in fatty acid synthesis (Wang et al., 2020) and phospholipid membrane remodelling (Kraft et al., 2020). BH4 is also important in regulation of ether lipid metabolism via alkylglycerol monooxygenase by cleaving the ether bond of alkylglycerols (Watschinger et al., 2015). The present study has shown that *Gch1*/BH4 could also play an important role in fatty acid catabolism by regulating the expression of some key enzymes involved in fatty acid metabolism and oxidation in mitochondria. The present data demonstrated that the downregulation of fatty acid oxidation enzymes, possibly caused by a reduction in mitochondrial number, drives fatty acid away from catabolic pathway and into the formation of intracellular TAG. Kim et al. (2019) has shown that BH4 deficiency in the global sepiapterin reductase (*Spr*) KO mice model decreases transcription of major mitochondrial biogenesis regulatory genes, including *Ppargc1a*, *Ppara*, *Esrra* and *Tfam*, but these genes were not significantly altered in cm*Gch1* KO mice (data not shown). This suggests that an alternative mechanism is involved for *Gch1*/BH4 in the regulation of lipid metabolism, or that the cardiomyocyte‐specific KO of *de novo* BH4 synthesis in cm*Gch1* KO mice is less extreme than the very severe systemic phenotype of the global *Spr* KO mouse. Other mouse models of BH4 deficiency with either reduced pyruvoyltetrahydropterin synthase activity (*Pts* mutant) or *hph‐1* mice also showed alterations in energy metabolism associated with obesity, glucose intolerance and insulin resistance (Korner et al., 2016; Oguri et al., 2017). Likewise, transcriptome analysis in *Pts* mutant animals indicated changes in glucose and lipid metabolism, suggesting that BH4 regulates cell‐specific cardiac metabolism as well as systemic metabolism.

Preclinical studies of acute myocardial infarction suggest that lethal reperfusion injury accounts for up to 50% of the final cardiac infarct size (Yellon & Hausenloy, 2007). The metabolic changes in fatty acid oxidation in cm*Gch1* KO mice are associated with striking protective effects in response to myocardial IR injury and infarct size. These phenotypes were unlikely induced by prolonged high‐level expression of Cre recombinase as there were no differences observed between α‐MHC Cre^+/−^ and *Gch1^flox/flox^* mice. Fatty acid metabolism requires high oxygen consumption, which is not available during ischaemia, such that the metabolic changes in cm*Gch1* KO hearts may provide metabolic adaptations that enable the heart to mitigate IR injury by favouring glycolysis. Furthermore, a transient hyper‐contracture is normally observed at the initial reperfusion due to calcium overload and other cellular processes, which can lead to irreversible tissue damage and contractile dysfunction (Hausenloy & Yellon, 2013). We observed that cm*Gch1* KO hearts showed no evidence of hyper‐contracture in the early reperfusion phase, and superior contractile function thereafter. This observation suggests that cm*Gch1* KO hearts have an early protective mechanism in response to the immediate impact of reperfusion. The reduced levels of long‐chain fatty acid in cm*Gch1* KO hearts at the beginning of reperfusion may contribute to these protective mechanisms. Previous studies have shown that addition of palmitate at reperfusion reduces cardiac function (Johnston & Lewandowski, 1991) and inhibition of fatty acid metabolism enhances glucose oxidation and improves contractile function in reperfused myocardium (Hendrickson et al., 1997; Lopaschuk et al., 1990). These observations support the notion that reduced fatty acid metabolism while upregulating glycolysis in the cm*Gch1* KO heart protects against IR injury. Indeed, the diabetic heart has increased susceptibility to IR injury because of decreased myocyte glucose uptake and utilisation that lead to impairment of ATP production by glycolysis during ischaemia, as well as toxic accumulation of free fatty acids and their oxidation product (Kota et al., 2011). Thus, targeting myocardial metabolism in diabetic heart via BH4 could be beneficial, as we previously demonstrated in a cardiomyocyte‐specific *Gch1* transgenic mouse, where augmentation of cardiomyocyte BH4 maintained cardiac function, glucose uptake and utilisation in diabetes (Carnicer Hijazo et al., 2021).

### 4.1 Conclusion

The present study has demonstrated that *Gch1* and BH4 regulate fatty acid oxidation in cardiomyocytes via alterations in glycolysis and fatty acid metabolism. The altered fatty acid oxidation in mice with cardiomyocyte BH4 deficiency protects against IR injury, suggesting that manipulating cardiac metabolism at reperfusion is cardiac protective.

## AUTHOR CONTRIBUTIONS

This study was conducted at the University of Oxford. Sandy Chu, Mark Crabtree, Gillian Douglas, Lisa Heather and Keith Channon have contributed to the study design, analysis and interpretation of the data. Jennifer K. Bendall has developed the cm*Gch1* KO model. Sandy Chu, Surawee Chuaiphichai and Thomas Nicol have conducted the experiments, and Benjamin Wright, Matthieu Miossec have done the RNAseq analyses. The manuscript was drafted by Sandy Chu. All authors have critically revised the important intellectual content of the paper and approved the final version of the manuscript. All authors agree to be accountable for all aspects of the work in ensuring that questions related to the accuracy or integrity of any part of the work are appropriately investigated and resolved. All persons designated as authors qualify for authorship, and all those who qualify for authorship are listed.

## CONFLICT OF INTEREST

The authors declare no conflicts of interest.

## Supporting information


Statistical Summary Document

## Data Availability

Data supporting the findings of this study are available from the corresponding author upon reasonable request. RNA‐seq dataset is available on Gene Expression Omnibus data repository; database accession number: GSE206063 (https://www.ncbi.nlm.nih.gov/geo/query/acc.cgi?acc=GSE206063).

## APPENDIX A1

### A1.1 Supplementary methods

#### A1.1.1 Excision allele detection

To confirm the deletion of LoxP sites in cardiomyocyte from cm*Gch1* KO mice, total DNA was extracted from different tissue and cells using Qiagen DNeasy blood and tissue kit (cat. no. 69504), followed by PCR using primer sequence stated in Table A1. A *Gch1* knocked‐out allele was identified at band position of 1300 bp, and a floxed allele without excision has a band at 1030 bp.

#### A1.1.2 Genotyping

Genomic DNA was prepared from ear biopsies for PCR using the forward and backward primers stated in Table A1. *Gch1^flox/flox^* animals were identified by a band at 1030 bp. αMHC‐Cre positive mice displayed a band at 300bp with an internal control at a band of 200 bp.

#### A1.1.3 Real‐time qPCR

Animals were anaesthetised using inhaled isoflurane and organs were excised. Total RNA was extracted from the cells and tissues using Qiagen RNeasy Mini Kit (cat. no. 74106) according to the manufacturer's instructions with on‐column DNase step. RNA was quantified using a Nanodrop spectrophotometer. RNA from cardiomyocytes (500 ng) and RNA from organ tissues (1000 ng) were converted to cDNA using the Qiagen QuantiTect Reverse Transcription Kit (cat. no. 205311) according to the manufacturer's instructions. All cDNA samples were diluted to 2 ng/μl and 5 ng was used for qPCR per reaction along with the *Gch1* Taq‐man assay primer (cat. no. Mm00514993) and Taq‐man PCR universal mix (Thermo Fisher Scientific). β‐Actin was used as an internal control for each sample and the relative quantification of *Gch1* expression between WT and cm*Gch1* KO was determined by $2^{-\Delta \Delta C_{T}}$ method, where ΔΔ*C*_T_ is the fold change relative to WT.

#### A1.1.4 Blood pressure measurements

Conscious blood pressure from 16‐week‐old WT and cm*Gch1* KO mice was obtained by using Visitech tail‐cuff BP‐2000 plethysmography system (Visitech, North Carolina, USA). Mice were trained for five consecutive days followed by 3 days of measurements. All blood pressure recording were taken at 09.00–12.00 h to avoid blood pressure fluctuation throughout the day. The systolic blood pressure was averaged from the 3 days’ measurement.

#### A1.1.5 Mitochondrial DNA quantification

DNA were extracted from left ventricular tissue of the mice using Qiagen DNeasy blood and tissue kit (cat. no. 69504) according to the manufacturer's instructions. The extracted DNA was quantified by Nanodrop and adjusted to 10 ng/μl with nuclease‐free water. mtDNA copy number was quantified by two nuclear encoded genes (*Actb* and *Adha*) and two mitochondrially encoded genes (*Cytb* and *mtCo2*). qPCR was performed with 20 ng of DNA, 5 μl of 2× iTAQ Universal SYBR Green Supermix (Bio‐Rad), and 0.06 μl of the relevant forward and backward primers at 50 μM concentration. The final volume was adjusted to 10 μl with nuclease‐free water. qPCR was performed with initiating step at 95°C for 5 min, followed by 40 cycles of 95°C for 5 s and 60°C for 30 s. mtDNA copy number were calculated by averaging 2^Δ^ *^C^* ^T^ of *Actb* versus *Cytb*, and *sdha* versus *mtCo2*.

#### A1.1.6 Mitochondrial isolation and respiration

Mice were terminally anaesthetised using inhaled isoflurane and the hearts were excised into ice‐cold solution A containing 250 mM sucrose, 0.5 mM Na_2_EDTA and 10 mM Tris, at pH 7.4. The heart tissues were homogenised in solution A (1 ml per 100 mg tissue) and trypsin (0.1 ml per 100 mg tissue) was then added and incubated on ice for 10 min for further homogenisation. After incubation, solution B (1 ml per 100 mg tissue) containing solution A and 0.5% BSA was added into the tissue homogenate and centrifuged at 590 *g* at 4°C for 10 min. The supernatant was transferred into a new 15 ml Falcon tube and centrifuged at 3020 *g*, 4°C for 10 min. The cell pellets were resuspended with solution B (0.5 ml per 100 mg tissue) and the centrifuge protocol were repeated twice. The final cell pellets which contained the isolated mitochondria were suspended in 0.15 ml KME solution (100 mM KCl, 50 mM MOPS and 0.5mM EGTA) and protein concentrations were determined (BCA protein assay detection kit, Thermo Fisher Scientific).

Mitochondrial respiration was measured using a Clark‐type oxygen electrode (Strathkelvin Instruments Ltd, North Lanarkashire, UK) at 30°C, as previously described (Heather et al., 2012). Mitochondria were incubated in 0.5 ml respiration medium (100 mM KCl, 50 mM MOPS, 1 mM EGTA, 5 mM KH_2_PO_4_, and 1 mg/ml BSA, pH 7.4) and oxygen consumption was measured using pyruvate (10 μM) with malate (5 mM), palmitoyl‐CoA (40 μM) with carnitine (5 mM) and malate, and palmitoyl carnitine (40 μM) with malate. State 3 respiration was stimulated by the addition of 100 nmol of ADP, and subsequently state 4 was measured, which occurs when all the ADP added to the respiration medium had been phosphorylated. State 3 and 4 measurements were repeated after addition of a further 100 nmol of ADP, before a maximum rate of respiration was measured following addition of carbonyl cyanide 4‐(trifluoromethoxy) phenylhydrazone (1 μM).

### A1.2 Tables and figures

**TABLE A1:** Primers used to genotype transgenic mice.

PCR Primers	Sequence 5′‐3′
*Gch1* excised allele	GCTCATCCCCCACACTTGTCT
*Gch1*	Forward: GTCCTTGGTCTCAGTAAACTTGCCAGG
	Reverse: GCCCAGCCAAGGATAGATGCAG
*α‐MHC Cre*	Forward: ATGACAGACAGATCCCTCCTATCTCC
	Reverse: CTCATCACTCGTTGCATCATCGAC
WT control reverse	Forward: CAAATGTTGCTTGTCTGGTG
	Reverse: GTCAGTCGAGTGCACAGTTT
*Gch1* Taqman assay	Mm00514993_m1

**TABLE A2:** Genes associated with fatty acid metabolism analysed by Cytoscape are listed, along with the corresponding fold change, cmGch1 KO vs. WT, found in the RNA sequencing data.

Gene name	Gene description	Fold change (Log)
Gpcpd1	glycerophosphocholine phosphodiesterase GDE1 homolog	−0.592
Acsl6	acyl‐CoA synthetase long‐chain family member 6	−0.441
Mid1ip1	Mid1 interacting protein 1 (gastrulation specific G12‐like	−0.43
Ech1	enoyl coenzyme A hydratase 1, peroxisomal	−0.354
Hahd	hydroxyacyl‐Coenzyme A dehydrogenase	−0.348
Acacb	acetyl‐Coenzyme A carboxylase beta	−0.322
Acads	acyl‐Coenzyme A dehydrogenase, short chain	−0.321
Eci1	enoyl‐Coenzyme A delta isomerase 1	−0.304
Dgat2	diacylglycerol O‐acyltransferase 2	−0.292
Acss1	acyl‐CoA synthetase short‐chain family member 1	−0.277
Pccb	propionyl Coenzyme A carboxylase, beta polypeptide	−0.273
Pcca	propionyl‐Coenzyme A carboxylase, alpha polypeptide	−0.268
